# Computational modeling of cotranscriptional RNA folding

**DOI:** 10.1016/j.csbj.2025.06.005

**Published:** 2025-06-11

**Authors:** Lei Jin, Shi-Jie Chen

**Affiliations:** Department of Physics and Astronomy, Department of Biochemistry, Institute of Data Sciences and Informatics, University of Missouri, Columbia, MO 65211, USA

**Keywords:** RNA folding, Cotranscriptional folding, Computational modeling, Kinetic pathway, Structure prediction

## Abstract

An RNA folds as it is transcribed. RNA folding during transcription differs fundamentally from thermodynamic folding. While thermodynamic folding reaches an equilibrium ensemble of structures, cotranscriptional folding is a kinetic process where the RNA structure evolves as the chain elongates during transcription. This dynamic folding pathway causes cotranscriptional structures often to deviate from thermodynamic predictions, as the system rarely reaches equilibrium. Since these cotranscriptional effects can persist in the mature RNA's structure, understanding this kinetic process is crucial. While experimental studies of cotranscriptional folding have been successful, they remain resource-intensive. Computational modeling has emerged as an increasingly practical and powerful approach for investigating these dynamics. This short review examines current computational methods and tools for simulating cotranscriptional folding, with the goal of advancing our understanding of RNA folding mechanisms.

## Graphical abstract

**Figure:**
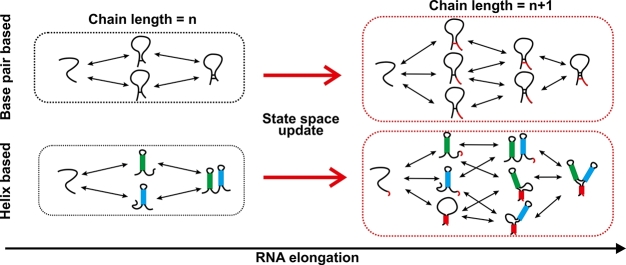

## 1 Introduction

RNA folding occurs primarily during transcription in cellular environments [1], [2], [3], [4]. This cotranscriptional folding process is precisely encoded within RNA sequences [5]. Understanding both the secondary and tertiary structures of RNA molecules in cells has become a central focus of RNA structural biology research, as these structures directly determine the biological functions of many RNAs, particularly non-coding RNAs [6], [7], [8], [9], [10]. Recent experimental evidence has demonstrated that mature RNAs transcribed both *in vivo* and *in vitro* retain substantial structural features that originate during cotranscriptional folding [11], [12].

Many gene expression-regulatory non-coding RNAs (ncRNAs), particularly riboswitches, execute their biological functions through structures formed during cotranscriptional folding and its intermediates [13], [14], [15], [16], [17], [18]. The kinetic nature of this process—where nascent RNA chains often lack sufficient time to reach equilibrium before new nucleotides emerge from RNA polymerase—means that cotranscriptionally formed structures, rather than equilibrium structures, typically provide more accurate insights into RNA functionality [8].

During cotranscriptional folding, the growing RNA chain explores diverse transient intermediates, potentially adopting final structures that differ significantly from the thermodynamically optimal structure of the full-length RNA [4], [8], [19], [20]. The interplay between transcription and RNA folding is modulated by multiple factors, including ionic conditions, metabolite presence, transcription rate, and site-specific polymerase pausing. These factors create complex folding pathways and can dramatically influence the final structural outcome [21], [22], [23], [24], [25].

Several experimental techniques have been employed to investigate nascent RNA structural landscapes during cotranscriptional folding [26]. Next-generation sequencing (NGS)-based methods, such as SPET-seq [11], eSPET-seq [12], and TECprobe-VL [27], enable systematic tracking of RNA cotranscriptional folding. These advanced approaches provide step-wise information on RNA secondary structure along the transcription timeline. However, due to inherent limitations of probing techniques (e.g., challenges in capturing rare intermediates or high-resolution 3D structures), reconstructing the complete details of every cotranscriptional intermediate from experimental data alone remains a challenge [28].

Moreover, high-throughput experimental methods can be time-consuming and costly, particularly when investigating large numbers of RNAs or long sequences. As a result, various *ab initio* computational models for RNA cotranscriptional folding have been developed [29]. These models predict partial or complete folding pathways using energetic and kinetic frameworks, often with minimal or no experimental constraints. Consequently, they serve as valuable tools for understanding mechanistic details and refining hypotheses for subsequent experimental validation.

This review presents an overview of computational methods and tools for modeling RNA cotranscriptional folding, with and without the integration of experimental constraints. We emphasize the key features of core algorithms, highlight notable achievements and applications, and discuss how computational approaches can deepen our understanding of RNA folding dynamics. This compilation aims to serve as a useful resource for researchers investigating RNA cotranscriptional folding mechanisms and structure–function relationships through computational means.

## 2 Computational predictions of cotranscriptional folding kinetics, intermediates, and pathways

**Fig. 1:**
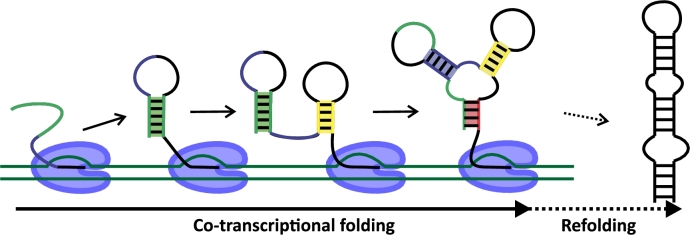
An illustration of RNA folding during transcription in the cell. As the RNA chain elongates, three kinetic intermediates form sequentially, each containing metastable helices shown in color. Some intermediates, such as a three-way junction, may be biologically functional prior to the RNA refolding into its thermodynamically stable structure, such as the extended stem-loop.

RNA folding can be described from multiple perspectives, ranging from a purely thermodynamic view of stable secondary structures to a dynamic, stepwise process influenced by kinetic trapping and detrapping [30]. Thermodynamic models typically identify the minimum free energy (MFE) structure or explore an ensemble of structures weighted by the Boltzmann factor. However, these approaches assume that RNA can sample all available conformations and eventually reach either the global thermodynamic minimum or an equilibrium ensemble. While thermodynamic considerations are essential for estimating the relative stability of base-pairing patterns, RNA folding in biologically relevant contexts is often far from equilibrium due to limited sampling time, macromolecular crowding, and the continuous elongation of the nascent transcript. For example, as shown in Fig. 1, RNA can cotranscriptionally fold through a series of non-equilibrium kinetic intermediates, forming functional structures such as a three-way junction, before eventually refolding into a thermodynamically stable conformation, such as an extended stem-loop.

### 2.1 Thermodynamics vs. kinetics in cotranscriptional folding

From a thermodynamic perspective, the RNA folding landscape can be visualized as a high-dimensional surface of free energy versus possible conformations [30]. While the MFE structure may be the most stable at equilibrium, RNA can become kinetically trapped in suboptimal structures if transition barriers to the global minimum are too high or if the elongating chain fails to sufficiently explore conformational space [30], [31], [32]. Unlike most *in vitro* conditions, the cellular environment does not always permit complete thermodynamic refolding due to constraints on solution conditions and timescales. Consequently, in-cell matured RNA molecules could adopt alternative structures distinct from those formed *in vitro*. Thus, strictly equilibrium-based predictions often fall short in capturing the diversity of RNA structures observed in the cellular context.

Kinetic models, however, describe RNA folding as a time-dependent process, where early-formed base pairs can constrain subsequent folding by preventing alternative pairings. This can lead to stable, long-lived structures that persist throughout the RNA's functional lifetime. Kinetic barriers, such as the rearrangement of base pairs required for transitions between distinct helices, often dictate the folding pathway.

### 2.2 Cotranscriptional folding algorithms and parameterization

To model RNA folding kinetics in cells, various computational algorithms have been developed. These approaches either track RNA conformations at each transcription length or model the full-length RNA as a dynamic system that stochastically transitions between local minima over time. One widely used strategy involves representing the energy landscape as a kinetic network, where local minima are connected by transition states. This framework has been applied in a broad range of systems, enabling researchers to map plausible folding pathways [33], [34].

Since the kinetic network can be fully described by the Master Equation, directly solving the equation becomes a straightforward approach to characterizing RNA folding kinetics. However, solving the Master Equation computationally is challenging due to the large number of conformational states included in the equation, particularly for longer RNA sequences. An alternative approach is to use kinetic Monte Carlo simulations, which approximate numerical solutions for complex kinetic networks. In general, computational modeling of RNA folding kinetics relies on the following key factors:

• **Free energy rules:** Most models use the Turner energy rules [32] or their variants to estimate secondary structure stability. For tertiary interactions, more complex force fields or knowledge-based potentials may be incorporated.
• **Conformational sampling:** Some methods explicitly enumerate all the possible base-paired conformations (secondary structures) of a given sequence, while others use Monte Carlo or coarse-grained simulations to stochastically explore conformational space.
• **Folding speed and transition rates:** The rate at which one structure transitions to another depends on energy differences and, in some models, an estimated activation barrier. Simple approaches use rate constants proportional to $\exp(-\Delta G/k_{B}T)$, where Δ*G* is the free energy difference between the two structures, whereas more advanced methods explicitly calculate energy barriers between distinct secondary or tertiary structures.

For example, methods such as Kinfold [35] simulate RNA folding by generating short trajectories of secondary structure transitions, using a Monte Carlo simulation to stochastically jump between adjacent structures in base-pair space. Similarly, the Gillespie algorithm [36] can be applied to kinetic modeling by defining a set of allowed base-pairing transitions along with their corresponding rates.

Among the three key components of a kinetic model, the parameterization of transition rates is crucial. In kinetic models, the state space is often coarse-grained, making transitions between states effectively multi-step processes. For instance, Zhang and Chen employed a zip-like downhill folding model [37], [38], [39] to estimate helix folding rates, whereas Xu, Yu, and Chen used kinetic Monte Carlo simulations [40], [41] to compute overall transition rates for helix formation.

Molecular dynamics (MD) simulations have been applied to study RNA kinetic folding at the 3D level. However, due to the high computational cost, traditional all-atom MD simulations have been primarily limited to base-pair-level investigations [42], providing the detailed kinetic profiles. Advanced molecular dynamics (MD) techniques, such as Targeted Molecular Dynamics (TMD), facilitate large-scale conformational sampling and have been used to explore kinetic transition pathways between distinct RNA structures [43]. To improve efficiency, coarse-grained models combined with appropriate force fields can replace all-atom representations, enabling the simulation of RNA kinetic folding for longer sequences while maintaining computational feasibility.

These computational approaches (summarized in Table 1) offer insights into the complexity of RNA folding kinetics, including phenomena such as misfolding, kinetic trapping, and detrapping of intermediate states. They also serve as a foundation for studying cotranscriptional folding, where sequence length and base-pairing probabilities continuously expand as transcription progresses.

**Table 1:** Computational methods for RNA folding kinetics.

Basic Method	RNA Length	Speed	Key Advantages	Main Limitations
Master equation	Short to medium	Moderate	High precision, relatively efficient	Require full knowledge of kinetic network, limited scalability
Stochastic simulation	Short to medium	Slow	High precision, sampling kinetic network	Difficulties in convergence for long sequence, high computation cost
Kinetic Monte Carlo	Medium to long	Fast	Enhanced efficiency in kinetic network sampling	Require rescaling for time-space, high computation cost
All-atom MD	Short	Slow	Atom-level precision, all information included	Require 3D structures, extremely high computation cost
Coarse-grained MD	Long	Variable	Balanced between precision and efficiency	Require 3D structures, incomplete kinetic information

## 3 Cotranscriptional folding

Cotranscriptional folding involves the diverse and dynamic structural states an RNA transcript adopts as it elongates. As RNA polymerase moves along the DNA template, the nascent RNA progressively extends, altering possible base-pairing interactions and revealing new structural motifs. This sequential folding process has profound implications:

• Early-formed structures can persist throughout transcription unless disrupted by helicases or other specific proteins, or a strong thermodynamic or kinetic drive to refold.
• Transcriptional pauses, regulated by proteins and specific RNA sequences, provide time windows for the partial transcript to explore alternative conformations.
• The emergence of structural motifs (e.g., hairpins, pseudoknots) at different transcript lengths can influence downstream folding pathways and structural transitions.

Thus, the final folded structure of a full-length RNA may differ from predictions based solely on equilibrium models or kinetic algorithms applied to the complete sequence. Instead, the transcript's elongation history and transient intermediate structures can guide it into unique kinetic traps or functional conformations.

Below, we outline two broad categories of computational approaches for modeling cotranscriptional folding: (1) *Ab initio* methods, which rely solely on energetic and kinetic principles (physical theories describing RNA folding thermodynamics and kinetics) and (2) Experiment-assisted strategies, which integrate empirical structural data (e.g., chemical probing or NGS-based structure mapping) into the simulation framework. See Table 2 for a summary of the reviewed models.

**Table 2:** RNA Cotranscriptional Folding Models.

Model	Method description	RNA length applicability	Advantages	Limitations
CoFold [45]	Predicts RNA folding incorporating cotranscriptional constraints	Medium to long	Simple and fast for long RNA	Indirect kinetic treatment, cannot predict folding pathway
Kinwalker [48]	Stepwise thermodynamically optimal folding trajectories	Long	Applicable for long RNA	Non-kinetic, thermodynamic folding
Kinfold [35]	Stochastic simulations with single-base-pair moves	Short to medium	Kinetic folding at single base pair resolution	High computation cost, chain elongation not considered
Ndifon's model [49]	Stochastic selection of structural rearrangements	Short to medium	Captures non-linear RNA folding dynamics	Rapid increased computation cost, chain elongation not considered
Kinefold [29], [50], [51]	Kinetic Monte Carlo, single base-pair transitions	Short to medium	Tracks dynamic folding pathways including pseudoknots	Intensive computation cost for long sequence
CoStochFold [52]	Stochastic simulation integrated with transcription dynamics	Short to medium	Captures realistic folding dynamics	High computation cost
RNAkinetics [53]	Probabilistic ensemble analysis with helix-based kinetic modeling	Short	Comprehensive probabilistic folding pathways	Unreliable rate calculation, limited scalability for various RNAs
Zhao's model [55], [56], [57]	Analytical stepwise predictions with helix-based kinetic modeling	Short to medium	Accurate transition rate, detailed folding pathway	Challenges with highly complex structures
Sun's model [59]	Helix-based kinetic modeling	Short to medium	Accurate transition rate, pseudoknot considered	Limited to helix-based conformation sampling
Xu's model [60]	Helix-based kinetic modeling, multiscale energy landscape approach	Medium	Balances accuracy and computation cost, improved rate calculation accuracy	Challenging for large RNAs with extensive conformational diversity
DrTransformer [62]	Heuristic ensemble approach to RNA folding	Medium to long	Handles large RNAs efficiently	Accuracy depends on structural diversity and chosen parameters
Cotranscriptional SHAPE-seq [16]	Integrates experimental probing with computational analysis	Short to medium	Experiments and bioinformatics pipeline	Multi-step computational analysis required
R2D2 [83]	Integrates high-resolution SHAPE-seq data with advanced structure prediction	Short to medium	Efficient interpretation of experimental data	Rely on experiment quality, cannot distinguish alternative folding pathway
TECprobe-VL [27]	Integrates streamlined experimental and computational approaches to map RNA folding pathways	Applicable for longer sequences	Multi-step computational analysis required	

### 3.1 *Ab initio* modeling

*Ab initio* modeling of RNA cotranscriptional folding attempts to predict folding pathways based solely on thermodynamic and kinetic parameters, without incorporating direct experimental structural data. These methods vary in their level of detail regarding dynamic simulation, energetic modeling, and structural complexity (e.g., secondary vs. tertiary structure). Below, we review commonly used *ab initio* methods and tools for cotranscriptional folding.

#### 3.1.1 Stepwise elongation algorithms

A straightforward approach to modeling cotranscriptional folding is stepwise elongation simulation. At each step, a nucleotide is appended to the 3' end of the growing RNA chain, followed by secondary structure prediction using methods such as minimum free energy estimation or suboptimal structure sampling for the newly extended transcript.

The ViennaRNA package offers features such as “–noLP” (to exclude lonely base pairs) and “–shape” (to incorporate structural constraints) for simulating cotranscriptional folding by generating the MFE structure at each transcription step. While primarily thermodynamic equilibrium in nature, it can be supplemented with kinetic modeling tools, such as the *barriers* program or Kinfold, to account for folding dynamics [44].

COFOLD [45] is a novel RNA secondary structure prediction method that explicitly accounts for cotranscriptional folding effects. COFOLD addresses cotranscriptional folding effects by incorporating a scaling function that modifies energy contributions based on the nucleotide distance between potential pairing partners. This function models the decreased likelihood of long-range interactions during cotranscriptional folding. As a result, COFOLD improves prediction accuracy, particularly for long RNA sequences exceeding 1000 nucleotides, including rRNAs. By combining established energy models [46], [47] with kinetic considerations, COFOLD achieves higher sensitivity, specificity, and overall performance compared to predictions without kinetic considerations. The method thus provides more biologically relevant structure predictions without substantially altering free-energy values [45]. An extension to the RNAfold approach attempts to handle cotranscriptional constraints more explicitly, such as considering certain structural features that can form prematurely and remain stable. However, the indirect kinetic treatment of cotranscriptional constraints can fail to capture the realistic dynamics of RNA folding and, as a result, cannot accurately reconstruct the corresponding folding pathway.

Kinwalker [48] is a heuristic method for simulating RNA cotranscriptional folding kinetics in large RNA molecules (up to about 1500 nucleotides). It combines thermodynamic predictions with kinetic modeling by precomputing locally optimal substructures using dynamic programming. These substructures, representing minimum free energy configurations of RNA segments, serve as building blocks for constructing complete folding pathways. The algorithm interleaves transcription and folding events to mimic cotranscriptional folding, where new nucleotides are sequentially added. It resolves conflicts between overlapping structures—those containing incompatible base pairs or substructures for dynamic programming algorithms—using energy-based criteria, and employs the Morgan–Higgs heuristic to estimate energy barriers (saddle points) along the folding pathway. Validated against experimental data from systems such as bacteriophage MS2, KU1, SV11, adenine-sensing riboswitches, and hok mRNA, Kinwalker reliably reproduces folding intermediates, kinetic traps, and refolding times, and offers valuable insights into the dynamic behavior of RNA folding. However, the locally optimal substructures used by Kinwalker can only form if local thermodynamic equilibrium is reached during transcription—a condition often violated in typical or extended transcriptional scenarios. Moreover, long-range interactions with downstream regions can destabilize these structures, leading to rearrangements. As a result, Kinwalker's reliance on local equilibrium limits its ability to accurately reconstruct cotranscriptional folding pathways. These stepwise approaches can be fast and straightforward to implement. However, they often only provide the MFE structure at each transcript length and might not account for the possibility that the chain is kinetically trapped in a non-MFE structure. As a result, the predicted final structure could be an oversimplification of the cotranscriptional pathway.

#### 3.1.2 Kinetic path simulation

Kinetic path simulation approaches explicitly track the dynamic evolution of RNA structure through a state-space of secondary structures during RNA chain elongation, typically employing a Master Equation or kinetic Monte Carlo methods. In these approaches, a kinetic folding model is applied for each step of the chain elongation (i.e., the time window for the transcribed chain before adding the next nucleotide). After each elongation step, the distribution in state space is updated according to the new nucleotide added. Such kinetic simulations are repeated until the RNA reaches its full length. The population distribution of the state space at each step is inherited from the previous step to keep the kinetic continuity (i.e., completeness of kinetic network and the populational continuity of each state).

**Fig. 2:**
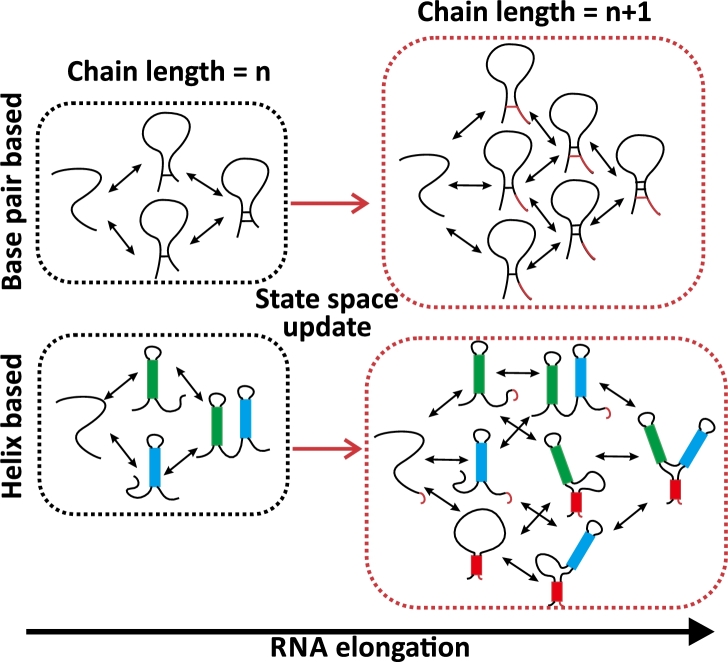
A comparative scheme of the basepair-based (upper route) and helix-based (lower route) kinetic path approaches to RNA cotranscriptional folding simulations. The simulated kinetic transition network is updated from left to right with RNA chain elongation by one nucleotide.

According to structural moves defined in the cotranscriptional kinetic model, current available kinetic path simulation methods can be classified into basepair-based kinetic simulation and helix-based kinetic simulation, see the illustration of the two approaches in Fig. 2. In the basepair-based kinetic simulation, the forming or breaking of a base pair is treated as a kinetic move, while in helix based kinetic simulation, the formation or deformation of an entire helix is treated as a kinetic move.

**Basepair-based Kinetic simulation**

Since an RNA secondary structure is represented by its base-pairing pattern, it is most convenient to treat base pair changes as kinetic moves in the simulation. In this approach, all possible RNA secondary structures can be considered as states in the kinetic network.

As part of the ViennaRNA software suite, Kinfold simulates RNA folding using a stochastic approach. Although not exclusively designed for cotranscriptional folding, it can be adapted by incrementally revealing nucleotides in a time-dependent manner. Kinfold enumerates neighboring structures in base-pair space, selecting the next state with probabilities governed by Boltzmann weights and transition rates [35]. These simulations yield a trajectory or ensemble of trajectories that can be analyzed to identify major folding pathways and intermediates. To enable Kinfold to generate folding pathways at single-base-pair resolution, its stochastic simulation algorithm requires extensive computational resources for pathway sampling. This high computational cost limits the method's applicability to short RNA sequences without pseudoknotted structures.

Ndifon developed a complex adaptive systems model for RNA kinetic folding [49]. The model views folding as a dynamic process driven by structural rearrangements at the secondary structure level. Each structural rearrangement, representing formation, dissociation, or shifting of individual base pairs, is assigned a fitness based on local stacking interactions. An autonomous stochastic process periodically (programmed repeating) selects a subset of these rearrangements for realization, ensuring that only mutually independent (with cross-linked base pairs excluded) moves occur concurrently. The model employs an extended pair-based move set and uses Metropolis-like transition probabilities. By capturing local interactions that have impact on the subsequent folding pathway, the approach predicts folding pathways including alternative stable and metastable states. The improved pathway sampling algorithm enhances computational efficiency for short RNAs and allows for finer resolution of kinetic folding pathways. However, it remains impractical for longer sequences due to the rapidly increasing computational time.

Based on the RNA kinetic folding model developed by Isambert et al. [29], [50], Kinefold is a web server and computational tool specifically designed for simulating the folding pathways of an RNA molecule during transcription through kinetic Monte Carlo and clustered stochastic simulations [51]. Kinefold models base-pair formation and dissociation using the Gillespie algorithm and allows for pseudoknot formation. It simulates transcription by elongating the RNA chain over time, either at a constant rate or following a user-defined schedule. Validated on diverse RNA types, including the pseudoknotted HDV genomic ribozyme, Kinefold captures the full cotranscriptional folding process, including chain elongation, branching pathways, kinetic traps, and partially stable intermediates. Unlike models that generate discrete folding pathways at fixed chain lengths, Kinefold continuously tracks dynamic folding throughout transcription, making it especially well-suited for studying RNA folding in biologically realistic contexts.

By integrating transcription as an explicit event in the kinetic simulation, the kinetic folding program StochFold [52] and its variant CoStochFold [52] allow the simulation to capture the dynamic interplay between transcription and folding, modeling the process as a competitive reaction between folding events governed by stochastic rates based on free energy changes and transcription events, which occur at a rate determined by the RNA polymerase speed. With an improved kinetic folding algorithm based on StochFold, CoStochFold achieves high accuracy with manageable computational cost for short to medium-length RNAs. When tested on E. coli SRP RNA and SV-11 RNA, CoStochFold produced results consistent with experimental observations for RNAs longer than 100 nucleotides.

The availability of free energy parameters, including both enthalpy and entropy, for RNA base pairing and stacking [31], [32], [46], [47] enables the calculation of rate constants for different transitions. However, the state space expands rapidly as the number of possible base-pairing configurations increases with chain length. Consequently, basepair-based methods are most effective for short RNAs due to the computational challenges posed by long sequence lengths.

**Helix-based Kinetic simulation**

Because an RNA secondary structure is built up by helices (i.e., stacks of base pairs) and helix formation is usually kinetically fast, using helix changes as the kinetic moves is an efficient way to reduce the size of the state space of a kinetic model.

RNAkinetics [53] models the secondary structure kinetics of an elongating RNA molecule via stochastic transitions between candidate helices. A candidate helix is defined as a non-extendable, complementary double-stranded segment. The model introduces two elementary kinetic moves: helix disruption and helix formation. Helix disruption is modeled with a rate constant proportional to the exponential in the helix free energy, while helix formation depends on the free energy change associated with loop closure. Using a kinetic Monte Carlo simulation, the model updates the RNA conformational state as the chain elongates, with time increments drawn from an exponential distribution. Multiple simulation runs yield a kinetic ensemble that provides probabilistic descriptions of folding pathways. An advanced version allows for overlapping helices via effective transition constants. However, helix-based transitions often involve multi-step and multi-route base-pair rearrangements [37], [41], [54], making the linear scaling method used in RNAkinetics to estimate transition rates unreliable. Although RNAkinetics significantly reduces computational cost for short to medium-length RNAs, the model has not been validated for more complex cases beyond tRNAs.

A model developed by Zhao et al. [55] systematically predicts RNA folding kinetics during transcription by treating chain elongation as a sequence of discrete steps, each corresponding to the addition of one nucleotide. At every step, the conformational ensemble of the nascent RNA is generated and its population kinetics is calculated using a master equation framework. Helices are treated as fundamental building blocks, with formation and disruption rates defined by Arrhenius-like expressions that incorporate free energy changes from stacking interactions. The model incorporates multi-route pathways for helix formation and refolding, along with an improved helix-based transition rate estimation algorithm. It also accounts for complex structural transitions, including tunneling pathways in which competing helices exchange base pairs without full unfolding. These enhancements improve the accuracy of helix-level transition rates and the modeling of transitions between competing helices. By integrating these kinetic steps over the entire transcription process, the model predicts cotranscriptional folding pathways, transition rates, and conformational populations, thereby revealing how transcription speed and local structural rearrangements dictate the eventual folding outcome. With continued improvements, such as a transition node approximation method to reduce the state space size [56] and algorithms that account for pseudoknots [57], this model can be applied to various systems including ribozymes and riboswitches. However, limitations remain. Estimation errors in transition rates can accumulate with the increasing number of helices [58], especially in larger and more complex structures, leading to reduced prediction accuracy. Additionally, the rapid expansion of the state space in such cases can significantly increase computational cost, which continues to limit the model's scalability to large or highly structured RNAs.

A recent helix-based kinetic model was developed by Sun et al. to predict cotranscriptional folding kinetics for riboswitches [59]. To efficiently treat the exponentially growing conformational space, the model introduces a conformational resampling algorithm (CRKR) that retains only kinetically important states (e.g., obligatory states on the pathway). Furthermore, to compare with the results from single molecule experiments, the model integrates force-dependent adjustments to the rate constants, enabling predictions of folding pathways and termination efficiencies under external tension for riboswitches. While the model uses a rate estimation method similar to that of the earlier work [58], it inherits the same limitations when applied to structurally complex RNAs. Specifically, the accuracy of rate predictions may diminish for large or highly structured RNAs due to cumulative estimation errors and unaccounted interactions.

More recently, Xu et al. introduced a “landscape zooming” model to predict RNA cotranscriptional folding kinetics [60]. This approach partitions the RNA folding energy landscape into discrete segments based on long, stable helices, grouping structurally similar conformations into “partitions” defined by different helix combinations. At a coarse-grained level, the model constructs an inter-partition kinetic network to capture slow folding transitions. For each transition, the model “zooms in” to simulate detailed kinetic trajectories of helix formation, disruption, and exchange at the base pair/stack level. This multiscale framework integrates master-equation–based population kinetics with a reduced conformational ensemble, enabling efficient prediction of folding pathways and kinetic intermediates during transcription. Applications to *E. coli* SRP RNA and HIV-1 TAR RNA demonstrate that the model accurately predicts experimental kinetics and provides new insights into conformational switching [61]. To broaden the application of this model, pseudoknotted structures should be considered in further development.

#### 3.1.3 Energy landscape-based models for cotranscriptional folding

An alternative to direct kinetic path simulation is to construct an “energy landscape” representation, where each node represents a local minimum in secondary structure space and edges connect minima that differ by a small set of base-pair changes. The *barriers* program from the ViennaRNA suite, for example, uses a flooding algorithm to identify local minima and the saddle points between them [34]. Once the global structure of the landscape is identified, a Master Equation or a rate matrix can be established to derive population kinetics among these minima.

DrTransformer [62] is a deterministic algorithm designed for simulating cotranscriptional RNA folding using the nearest neighbor energy model. DrTransformer captures the kinetics of RNA folding during transcription by extending the RNA sequence one nucleotide at a time. At each step, it identifies new candidate structures via constrained folding in the exterior loop region, where the newly added nucleotide is available for pairing. The model constructs a guiding neighborhood—a minimal set of candidate transitions between structures—based on base-pair distance, defined as the cardinality of the symmetric difference between the base-pair sets of two structures. To estimate the transition rates, it calculates the energy barrier or “saddle energy” along direct paths between structures. To maintain computational efficiency, DrTransformer employs a top-down coarse-graining algorithm that reduces the ensemble to representative local minima, followed by kinetic simulations using rate matrices and matrix exponential methods. This approach closely approximates results from stochastic models such as Kinfold while significantly reducing computation time with high overall accuracy. However, due to limitations in its simulation algorithm, DrTransformer currently does not support pseudoknotted structures, which restricts its applicability to complex RNAs such as ribozymes and riboswitches, where pseudoknots often play an important role [63], [64], [65], [66].

Additionally, coarse-grained models such as oxRNA [67] treat each nucleotide as a reduced set of interaction sites. These models can be used for simulating both secondary and tertiary interactions in a physically motivated manner. Extensions to cotranscriptional folding systems include incrementally adding nucleotides to the chain and following the 3D structural dynamics. While computationally more expensive than secondary structure approaches, these models can incorporate important tertiary interactions such as helix stacking, coaxial interactions, or loop–loop contacts.

#### 3.1.4 Advantages and challenges

Computational modeling can provide an efficient and accessible means to investigate the mechanistic details of cotranscriptional RNA folding in silico. The ab initio models discussed above can predict or simulate folding pathways directly from RNA sequences, without relying on experimental constraints. This makes them well-suited for analyzing novel or large-scale datasets and for generating hypotheses about folding intermediates or structural regulators efficiently. Computational methods offer flexibility for testing the effects of transcription rates, ionic conditions, or synthetic sequence variants. However, these approaches also face certain challenges:

**Parameter accuracy**: Most current cotranscriptional folding models rely on accurate free energy calculations for RNA secondary structure. Despite decades of development, the thermodynamic parameters remain incomplete. While thermodynamic parameters for canonical base pairs (A–U, G–C, G–U) are well validated and widely applied in many RNA 2D structure energy model, (e.g., Turner rules) [32], parameters for non-canonical base pairs and loop segments—particularly long single-stranded loops—are based on estimates or extrapolations. Leading tools like RNAstructure [68], mFold [69], and Vfold [70] use approximated entropy parameters for these cases. Consequently, energy estimation remains a bottleneck, especially for complex motifs such as large junctions and pseudoknots.

**State-space exploration**: Simulating kinetic folding pathways requires a well-defined, complete set of conformational states. Due to the high dimensionality of RNA 3D structures, most ab initio models are limited to secondary structure representations. Even then, the number of possible conformations increases rapidly with RNA length. Exhaustively enumerating or simulating all possible kinetic states becomes impractical, necessitating the use of approximations or coarse-grained methods. As a result, models that simulate folding pathways are heavily dependent on efficient state-space exploration.

**Computational cost**: Given the potentially enormous state space for a given RNA sequence, fine-grained kinetic simulations are computationally intensive, especially for stochastic simulation for long RNAs. To manage complexity, many models adopt approximations, such as the MFE-based stepwise elongation method used in ViennaRNA [44]. However, such simplifications may not fully capture the complexity of folding kinetics.

**Prediction validation**: Validating cotranscriptional folding pathways remains difficult with current techniques. High-resolution direct measurements like X-ray crystallography and NMR are often unsuitable due to the short timescales involved (about 10 ms per kinetic step) [3], [71], causing real-time structure and pathway capture to become a major challenge. Indirect structure measurements like chemical probing [28] and cryo-EM [72] can be potentially capable of capturing RNA kinetic folding at certain timescales, however, the pathway interpretation approaches for such experimental data also remained unreliable.

To enhance the utility of computational models for RNA cotranscriptional folding, a balance between prediction accuracy and computational feasibility must be achieved. Multi-scale approaches, such as the partitioning strategy used in the Landscape-zooming model [60], offer a promising strategy. Parameter accuracy remains an intrinsic limitation. For example, thermodynamic parameters (enthalpy, entropy) are known to depend on environmental conditions such as temperature and ionic strength, reflecting a non-zero heat capacity change [31], [32]. This sensitivity can lead to significant shifts in folding pathways—even for simple structures like hairpins [54], [73], [74], [75], [76]. Addressing these issues may require case-specific parameter tuning that accounts for relevant environmental factors, rather than relying on generalized models. Some computational frameworks, such as the Poisson-Boltzmann methods [77], [78], TBI [79], [80], and MCTBI [81], allow for the rescaling of folding free energies under various ionic conditions. In parallel, single-molecule measurements (e.g., optical trapping to assess base-pairing/stacking energies) can offer more direct, condition-specific refinements [75], [76], [82]. Integrating computational models with experimental techniques can enhance the accuracy of transition rate calculations and improve the biological relevance of folding pathway predictions. Despite current limitations, computational methods remain indispensable tools for gaining physical insights into cotranscriptional RNA folding and for generating experimentally testable hypotheses.

### 3.2 Experiment-assisted modeling

Although *ab initio* approaches are valuable, computational methods alone may yield significant prediction uncertainties, particularly for RNAs that rely on delicate tertiary motifs or for those heavily influenced by cellular factors (e.g., proteins, specialized helicases). Experiment-assisted modeling attempts to reduce these uncertainties by integrating empirical structural information into the folding simulation. Below, we discuss emerging techniques and software that combine experimental data with computational prediction to achieve more accurate reconstructions of cotranscriptional folding pathways.

#### 3.2.1 Chemical probing constraints

A widely used approach in RNA structure determination is chemical probing. Notably, SHAPE (Selective 2'-Hydroxyl Acylation analyzed by Primer Extension), DMS (dimethyl sulfate), or CMCT (1-cyclohexyl-(2-morpholinoethyl) carbodiimide metho-p-toluenesulfonate) selectively modify flexible nucleotides (e.g., unpaired nucleoides), and by applying these probes at different stages of transcription (or for truncated constructs of various lengths), one may infer which regions of the RNA are single- or double-stranded at each partial length [28].

More recent technologies such as RING-MaP, SHAPE-Seq, or icSHAPE have advanced the resolution and throughput of chemical probing approaches. Experiment-assisted computational tools (e.g., with the “shape” option in the RNAstructure or ViennaRNA software) incorporate these reactivity data as pseudo–free energy constraints [44], [84] in structure prediction models. This strategy effectively biases the secondary structure prediction to be consistent with experimental reactivity profiles, resulting in structures that are more accurate than *ab initio* predictions.

For cotranscriptional folding, experimental techniques such as SPET-seq [11] and eSPET-seq [12] capture the structural signals at multiple nascent transcript lengths. Integrating these time-resolved or length-resolved data can be achieved by imposing constraints on nascent-length transcripts in a computational pipeline, thus refining the predicted pathway step by step.

#### 3.2.2 Structure probing during transcription

Several methods have been developed to probe RNA structure while transcription is in progress using immobilized transcription complexes or specialized polymerases to control pausing. For instance, TECPROBE and other variants [26], [27] freeze transcripts at specific lengths, allowing the user to collect chemical reactivity profiles or crosslinking patterns. Once these data are obtained, they can be integrated into modeling pipelines that systematically reconstruct the RNA conformational state at each paused length. Such datasets can reveal which base pairs remain stable over time, how structural rearrangements occur upon adding specific nucleotides, and whether alternative conformations or folding branches emerge.

Cotranscriptional SHAPE-seq [16] is a high-throughput method that integrates experimental probing with computational analysis to capture riboswitch folding at nucleotide resolution. The approach uses a library of DNA templates engineered with transcription roadblocks (transcription terminator) to generate halted RNA transcripts of defined lengths. Sequencing reads encode both the 3'-end position of the nascent RNA and the location of SHAPE-induced chemical modifications. The computational model processes these paired-end reads by binning them according to transcript length and computing SHAPE reactivity profiles for each nucleotide. This generates a reactivity matrix that captures dynamic structural transitions as the RNA is synthesized. Advanced bioinformatics pipelines perform quality control, alignment, and reactivity calculations to identify folding events such as hairpin formation, pseudoknot stabilization, and ligand-dependent bifurcation. By quantitatively mapping reactivity changes across transcript lengths, the computational framework links cotranscriptional folding pathways to the genetic regulatory function of riboswitches.

R2D2 [83] is a computational framework that reconstructs cotranscriptional RNA folding pathways by integrating high-resolution SHAPE-seq data (e.g., mutation profiles) with advanced structure prediction. It employs a sample-and-select approach that generates approximately 150,000 candidate secondary structures per nascent RNA length using stochastic sampling. A distance metric then compares predicted reactivity profiles with experimental SHAPE data to select the structures most consistent with observed folding intermediates. These selected structures form the basis for subsequent all-atom molecular dynamics simulations, where selective restraints guide 3D folding transitions. The multi-scale modeling pipeline (from RNA secondary structure to all-atom 3D structure) reveals rearrangement mechanisms such as toehold-mediated strand displacement that convert non-native intermediates into functional native folds. The method also evaluates the impact of point mutations on folding dynamics, providing mechanistic insights into RNA processing and regulation.

TECprobe-VL [27] is a novel cotranscriptional RNA structure probing method that integrates streamlined experimental and computational approaches to map RNA folding pathways with high resolution. The method employs a variable-length transcription elongation complex to capture nascent RNA structures as they emerge from RNA polymerase. On the computational side, the pipeline addresses biases inherent in sequencing coverage by implementing a data smoothing strategy, wherein reads from neighboring transcript lengths are concatenated to enhance effective depth and reproducibility. This computational model utilizes established tools, such as fastp (sequencing analyzing program) [85] for quality control, TECtools (a build-in NGS data processing program) for processing, and ShapeMapper2 (chemical probing data processing program) [86] for quantifying chemical reactivities, to generate precise reactivity matrices across transcript lengths. The resulting data, processed via overlapping window smoothing, enables accurate detection of coordinated folding events including hairpin formation, pseudoknot stabilization, and ligand-induced conformational changes. The integrated computational framework substantially improves the resolution and consistency of cotranscriptional RNA folding analyses.

#### 3.2.3 Combining single-molecule data and computational modeling

Another emerging approach is to combine single-molecule fluorescence or force spectroscopy data with computational modeling. Single-molecule Förster resonance energy transfer (smFRET), for instance, can track long-range interactions in RNA while it is transcribed [15]. Force-based methods such as optical tweezers can measure changes in the end-to-end distance of an RNA molecule under tension [87]. These techniques provide unique, time-resolved insights into folding transitions or misfolding events.

Incorporating single-molecule data into computational pipelines is an active area of research for RNA cotranscriptional folding. Typically, one might use a coarse-grained RNA model parameterized by standard free energy models, select conformations that match the observed smFRET efficiency distributions or force-extension curves. This synergy helps identify probable intermediate structures and refine the predicted kinetics of transitions.

#### 3.2.4 Advantages of experiment-assisted modeling

Combining in silico predictions with experimental data leads to several advantages:

**Improved accuracy**: Base-pairing configurations can be probed directly, albeit at varying resolution, through techniques such as chemical probing and NMR. When these experimentally derived pairing constraints are incorporated, they suppress spurious contacts and improve identification of true positive base pairs in computationally generated structures.

**Detection of alternative folds**: Experimental data can reveal structural heterogeneity through distinct signatures. When multiple signatures are observed for a given RNA region, modeling can incorporate alternative folding pathways and estimate their relative stabilities or population frequencies.

**Fig. 3:**
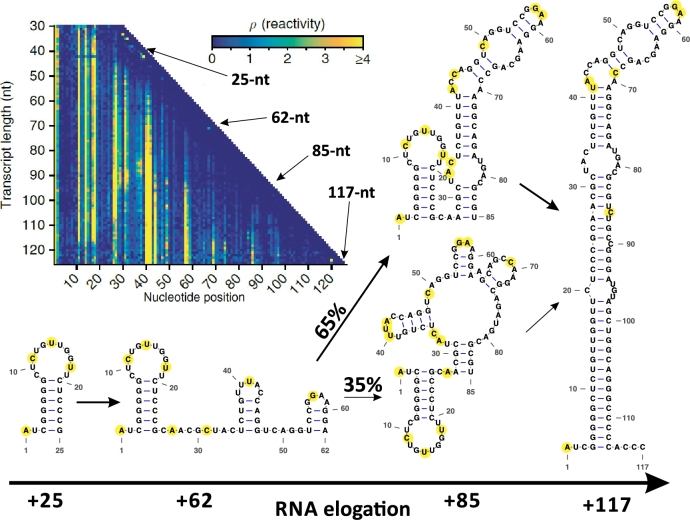
A practical example of reconstructing an RNA cotranscriptional folding pathway through the combined use of cotranscriptional chemical probing and computational modeling is shown here. The candidate structures and kinetic folding pathway were predicted using Vfold2D [70] and the landscape-zooming model [60]. Folding pathway population kinetics were inferred from cotranscriptional SHAPE experiments conducted by Watters et al. [16]. Experimentally identified unpaired nucleotides were highlighted in yellow on the predicted 2D structures, showing strong agreement with the computationally predicted secondary structures.

Despite these benefits, experiment-assisted approaches face important limitations. Single-molecule methods, while powerful, are technically demanding and can be difficult to interpret. Chemical probes typically produce ensemble-averaged signals [28], complicating analysis when multiple conformers (thus a mixture of signal profiles) coexist [88], [89]. Moreover, reagents such as SHAPE or DMS require reaction times of several minutes [28], during which structural refolding can occur, potentially obscuring true folding intermediates. Rapid-acting reagents like TMO shorten the reaction to a few seconds [90], reducing but not eliminating this problem and still struggle to capture the full cotranscriptional folding pathway due to their limited reactivity with certain nucleotides (e.g., G and U). Capturing an entire cotranscriptional folding pathway therefore remains challenging, and reproducing the native cellular context in vitro is even more so. These constraints underscore the need for tight feedback between computation and experiment. Although current techniques do not allow direct and complete observation of RNA cotranscriptional folding, they provide valuable kinetic and structural information that can be leveraged to improve computational models. In return, modeling improves the interpretation of experimental signals, enabling more accurate reconstructions of folding pathways. For instance, statistic or machine-learning frameworks that translate spectral features from cotranscriptional chemical probing into quantitative constraints can be merged with physics-based models to tackle large, complex RNAs, as an example shown in Fig. 3. Nonetheless, experiment-assisted modeling forms a crucial bridge between purely computational predictions and the intricate reality of RNA folding inside the cell.

## 4 Conclusion and perspectives

Cotranscriptional folding is fundamental to RNA biology, linking RNA structure to cellular function. Increasing experimental evidence suggests that an RNA's final structure is influenced not only by its sequence and thermodynamic stability but also by kinetic constraints and intermediate folds that form as the transcript emerges. More importantly, cotranscriptional RNA regulation is widely observed across all forms of cellular life [18]. From transient folding intermediates to the formation of native or misfolded structures in long transcripts, the interplay between transcription, structural transitions, and dynamic rearrangements is increasingly recognized as a key factor in regulating RNA functions such as splicing, activation, and degradation. For example, HIV-1 mRNA transcription is trans-activated by the Tat protein binding to the TAR domain in the 5' UTR, which folds cotranscriptionally at the very onset of transcription [61], [91]. In eukaryotes, cotranscriptional splicing is initiated by secondary structures that form at splice donor and acceptor sites during transcription [92], [93]. Additionally, various riboswitches rely on cotranscriptionally folded pseudoknotted structures to function as ligand-binding aptamers [63], [64], [65]. Understanding RNA cotranscriptional folding is therefore critically important for gaining a comprehensive view of RNA function within its cellular context.

In this review, we have summarized a broad range of computational strategies for modeling RNA cotranscriptional folding.

**Kinetic Folding Algorithms and Pathway Prediction:** We discussed the difference between thermodynamic (equilibrium) and kinetic perspectives, highlighting how RNA may become trapped in non-equilibrium conformations and how computational techniques track these folding pathways. Methods such as Kinfold and Kinefold illustrate the growing sophistication in modeling base-pair transitions under kinetic constraints.

***Ab initio* Cotranscriptional Folding:** Stepwise elongation methods and full kinetic simulations provide mechanistic insights into how partial transcripts might fold or misfold. Tools in the ViennaRNA package and coarse-grained models such as oxRNA offer a breadth of possibilities for systematically exploring folding pathways from first principles.

**Experiment-Assisted Modeling:** We emphasized the power of chemical probing approaches (SHAPE, DMS, etc.) and NGS-based methods (SPET-seq, eSPET-seq, etc.) for refining computational predictions. These data, integrated via pseudo–free energy terms or direct constraints, can significantly enhance the accuracy of structure modeling. Single-molecule techniques further reveal real-time folding events and can guide or validate computational pathways.

Future development of RNA cotranscriptional folding models may explore the following directions:

1. **Higher-order structures and beyond secondary structure:** While secondary structure modeling has reached a relatively mature stage, accurately capturing tertiary motifs such as pseudoknots and loop–loop interactions and global tertiary organization remains a significant challenge. Continued advancement in RNA 2D and 3D modeling depends heavily on improved energy parameter measurements, particularly for non-canonical base pairs, modified nucleotides, and tertiary interactions. Emerging integrative methods offer promising solutions by combining diverse experimental data sources: long-range base-pairing information from advanced chemical probing, short-range pairwise interactions from NMR, and global structural features from cryo-EM. These data can form a new foundation for cotranscriptional folding modeling. In particular, the ability of cryo-EM to capture three-dimensional structures of folding intermediates holds great potential for advancing cotranscriptional folding models beyond the secondary structure level, enabling a more comprehensive and accurate representation of RNA folding in biological contexts.
2. **Cellular environment:** RNA folding in vivo occurs in a crowded molecular environment, with the possibility of cofactors (ions, small molecules, RNA-binding proteins) significantly altering folding pathways. Future computational frameworks should increasingly incorporate ion conditions, protein-binding data, cotranscriptional epigenetic marks, and metabolic signals. Such integrative models could, for example, cooperate with most advanced RNA-ligand binding and RNA-RNA/protein docking, integrate theoretical model calculation and single-molecule experiment measurements to account for cellular-like conditions, and combine ribosome profiling data (for mRNA) with chemical probing to capture how translation and cotranscriptional folding might be coupled in prokaryotes.
3. **Modeling transcriptional pausing and speed variations:** Polymerase speed and site-specific pausing can influence the cotranscriptional folding landscape. Few modeling tools yet handle these processes in detail. For instance, different kinetic rates for elongation at specific sequence motifs can lengthen the time that partially formed RNA structures remain stable or form alternative folds. Combined with transcriptional pausing and elongation rate information extracted from various experiments including cotranscriptional chemical probing, developing robust computational frameworks that incorporate pause sites and elongation speed variation is crucial for accurately reproducing the real in vivo or in vitro transcription scenario.
4. **Integration of multi-omics data:** With the increasing availability of high-throughput approaches, multiple layers of information (transcriptome-wide structure probing, interactome data for RNA-binding proteins, etc.) are becoming accessible. Integrative modeling that combines these data streams can lead to a holistic picture of how RNA structures are orchestrated in the transcriptome.
5. **Machine learning and deep learning approaches:** Recent advances in protein structure prediction (e.g., AlphaFold) highlight the transformative potential of deep learning in structural biology. While RNA has unique challenges (e.g., strong base-pairing energies, more complex tertiary constraints, etc.), employing machine learning to predict the likely trajectory of cotranscriptional folding from large experimental datasets could be a game-changer. Model architecture that combine sequence features (e.g., local composition, GC content, or known motifs) with known structural signals could predict the kinetics of domain formation or misfolding events at the transcriptome scale. In integrating various and multi-omics data from experiments and computations, deep learning approaches can be most effective in reconstructing cotranscriptional folding pathway through multi-objective optimizations.

In summary, our understanding of cotranscriptional RNA folding has advanced significantly, driven by improved experimental methods and increasingly accurate computational tools. However, fully capturing the complexities of RNA folding in vivo remains a challenge, as transcription is intricately linked to protein-binding partners and other regulatory molecules. Integrating these factors into comprehensive computational frameworks, while refining thermodynamic and kinetic models, will be essential for developing a more complete picture of the interplay between RNA sequence, structure, and function.

## CRediT authorship contribution statement

**Lei Jin:** Writing – original draft, Methodology, Investigation, Formal analysis, Data curation. **Shi-Jie Chen:** Writing – review & editing, Supervision, Project administration, Investigation, Funding acquisition, Formal analysis, Conceptualization.

## Declaration of Competing Interest

None.
